# Phototrophic Co-cultures From Extreme Environments: Community Structure and Potential Value for Fundamental and Applied Research

**DOI:** 10.3389/fmicb.2020.572131

**Published:** 2020-11-06

**Authors:** Claire Shaw, Charles Brooke, Erik Hawley, Morgan P. Connolly, Javier A. Garcia, Miranda Harmon-Smith, Nicole Shapiro, Michael Barton, Susannah G. Tringe, Tijana Glavina del Rio, David E. Culley, Richard Castenholz, Matthias Hess

**Affiliations:** ^1^Systems Microbiology and Natural Products Laboratory, University of California, Davis, Davis, CA, United States; ^2^Bayer, Pittsburg, PA, United States; ^3^Microbiology Graduate Group, University of California, Davis, Davis, CA, United States; ^4^Biochemistry, Molecular, Cellular, and Developmental Biology Graduate Group, University of California, Davis, Davis, CA, United States; ^5^Department of Energy, Joint Genome Institute, Berkeley, CA, United States; ^6^Greenlight Biosciences, Inc., Medford, MA, United States; ^7^Department of Biology, University of Oregon, Eugene, OR, United States

**Keywords:** biodiversity, biotechnology, culture collection, cyanobacteria, extreme environments, fundamental research, microbial ecology and diversity

## Abstract

Cyanobacteria are found in most illuminated environments and are key players in global carbon and nitrogen cycling. Although significant efforts have been made to advance our understanding of this important phylum, still little is known about how members of the cyanobacteria affect and respond to changes in complex biological systems. This lack of knowledge is in part due to our dependence on pure cultures when determining the metabolism and function of a microorganism. We took advantage of the Culture Collection of Microorganisms from Extreme Environments (CCMEE), a collection of more than 1,000 publicly available photosynthetic co-cultures maintained at the Pacific Northwest National Laboratory, and assessed via 16S rRNA amplicon sequencing if samples readily available from public culture collection could be used in the future to generate new insights into the role of microbial communities in global and local carbon and nitrogen cycling. Results from this work support the existing notion that culture depositories in general hold the potential to advance fundamental and applied research. Although it remains to be seen if co-cultures can be used at large scale to infer roles of individual organisms, samples that are publicly available from existing co-cultures depositories, such as the CCMEE, might be an economical starting point for such studies. Access to archived biological samples, without the need for costly field work, might in some circumstances be one of the few remaining ways to advance the field and to generate new insights into the biology of ecosystems that are not easily accessible. The current COVID-19 pandemic, which makes sampling expeditions almost impossible without putting the health of the participating scientists on the line, is a very timely example.

## Introduction

Cyanobacteria are photosynthetic prokaryotes that are found in the majority of illuminated habitats and are known to be some of the most morphologically diverse prokaryotes on our planet (Whitton and Potts, 2000). The global cyanobacterial biomass is estimated to total ∼3 × 10^14^ g of carbon (Garcia-Pichel et al., 2003) and cyanobacteria may account for 20–30% of Earth’s primary photosynthetic productivity (Pisciotta et al., 2010). The efficient photosynthetic machinery of cyanobacteria has inspired growing interest in the utilization of cyanobacteria and cyanobacteria containing co-cultures in microbial fuel cells (Zhao et al., 2012; Gajda et al., 2015). In addition to having a global effect on the carbon cycle, cyanobacteria-mediated nitrogen fixation has been estimated to supply 20–50% of the nitrogen input in some marine environments (Karl et al., 1997). A detailed comprehension of cyanobacteria and their role in global carbon and nitrogen cycling is therefore indispensable for a multi-scalar and holistic understanding of these globally important nutrient cycles.

Besides their ecological relevance, cyanobacteria have potential applications in biotechnology: cyanobacteria facilitate the assimilation of carbon dioxide, a cheap and abundant substrate, to synthesize a variety of value-added compounds with industrial relevance (Al-Haj et al., 2016). Although monocultures have dominated in microbial biomanufacturing, controlled co-cultures have been recognized as valuable alternatives, due to their potential for reducing the risk of costly contaminations and in some cases enabling increasing product yield (Wang et al., 2015; Yen et al., 2015; Padmaperuma et al., 2018). Numerous cyanobacterial strains have been investigated for their potential to produce bioactive compounds, biofertilizer, biofuels, and bioplastics (Abed et al., 2009; Woo and Lee, 2017; Miao et al., 2018); and co-expression of non-cyanobacterial genes as well as co-cultivation of cyanobacteria with non-photosynthetic bacteria has resulted in self-sustained systems and improved desirable cyanobacterial phenotypes (de-Bashan et al., 2002; Subashchandrabose et al., 2011; Formighieri and Melis, 2016). Genes coding for enzymes capable of catalyzing reactions that result in unique products, such as modified trichamide, a cyclic peptide suggested to protect the bloom-forming *Trichodesmium erythraeum* against predation (Sudek et al., 2006); and prochlorosins, a family of lanthipeptides with diverse functions that are synthesized by various strains of *Prochlorococcus* and *Synechococcus* (Li et al., 2010; Cubillos-Ruiz et al., 2017), have been identified from cyanobacterial genomes (Zarzycki et al., 2013; Kleigrewe et al., 2016). It is very likely that *de novo* genome assembly from metagenomic data will facilitate the discovery of novel enzymes from cyanobacteria that are recalcitrant to current isolation and cultivation techniques. Although metagenome-derived genomes hold great potential to enhance our knowledge about genomic dark matter, improved techniques to isolate and enable axenic culturing of microorganisms that are currently considered as “unculturable,” as well as new genetic tools to study non-axenic cultures will be necessary in order to fully access the biotechnological potential of cyanobacteria.

Culture collections provide the possibility of preserving microbial isolates over extended periods of time without introducing significant genetic changes (McCluskey, 2017) and they provide easy access to these isolates and their associated metadata (Boundy-Mills et al., 2015). Although culture collections hold enormous potential for capturing and preserving microbial biodiversity, there are numerous challenges in maintaining these biological depositories. With recent advances in DNA sequencing technologies and the accessibility of 16S rRNA gene-based microbial community profiling, we are now well positioned to re-inventory, and standardize existing culture collections, which will be essential for preserving and cataloging the planet’s microbial biodiversity.

To explore the potential of culture collections, specifically those that maintain samples of microbial co-cultures, we reexamined the biodiversity of 26 historical phototrophic samples from the Culture Collection of Microorganisms from Extreme Environments (CCMEE). While some of the samples, selected for this project were studied previously (Supplementary Table 1) using cloned-based 16S rRNA profiling and morphological characterization (Camacho et al., 1996; Miller and Castenholz, 2000; Nadeau and Castenholz, 2000; Nadeau et al., 2001; Dillon et al., 2002; Dillon and Castenholz, 2003; Norris and Castenholz, 2006; Toplin et al., 2008), the diversity and the overall community assemblage of these co-cultures have not yet been characterized. We selected samples from environments with distinct and extreme physical properties from across the globe, suggesting each co-culture would yield a unique microbial consortium. Although reasonable to assume that these consortia have changed over time in composition and function (due to their cultivation), it is very likely that results obtained during this work will still provide insights into the microbial biodiversity of extreme habitats, some of which may no longer be accessible.

## Materials and Methods

### Sample Collection and Sample Description

Co-cultures selected for this study are part of a larger culture collection and were collected from different locations (Table 1) between 1988 and 2002. Isolates were collected using sterile techniques, kept in the dark and stored on ice as soon as possible. Samples were transported to the laboratory where aliquots were prepared preservation at −80°C and cultivation. For this study, co-cultures were selected from the CCMEE to cover a variety of geographical locations (Supplementary Figure 1) as well as a range of different ecosystems (Table 1). Due to the lack of a consistent usage of terminology to describe the sampling sites, we categorized co-cultures according to the geographical location (e.g., Antarctica, Bermuda, Denmark, Mexico and Spain) and based on the general description of the ecosystems (i.e., creek, crust, freshwater, hot spring, marine, saline pond, terrestrial, travertine, and tree bark) from where the co-cultures were collected. In addition, we used the growth medium and temperature (i.e., 12, 23, 40, 45, and 55°C) at which available co-cultures have been maintained by the CCMEE curators to categorize the selected co-cultures.

**TABLE 1:** Summary of photosynthetic co-cultures for which 16S rRNA gene profiles were generated.

**FECB ID^*a*^**	**CCMEE ID^*a*^**	**Growth temperature [°C]**	**Sample location [country]**	**Sample location [region]**	**Habitat**
FECB1	5011	12	Antarctica	McMurdo Ice Shelf; Bratina Island	Pond (saline)
FECB2	5019	12	Antarctica	McMurdo Ice Shelf; Bratina Island	Pond (freshwater)
FECB3	5034	12	Antarctica	McMurdo Ice Shelf; Bratina Island	Pond (brackish)
FECB4	5047	23	Spain	Lake Arcas	Lake (freshwater)
FECB5	5049	23	Spain	Lake Arcas	Lake (freshwater)
FECB6	5051	23	United States	Yellowstone National Park; Pott’s Basin	Hot Spring
FECB10	5056	23	Mexico	Viscaino Desert	Terrestrial (epilithic)
FECB12	5062-A	23	United States	University of Oregon; Eugene, Oregon	Terrestrial (concrete)
FECB14	5093	23	United States	Yellowstone National Park; Pott’s Geyser	Hot Spring
FECB15	5083	23	United States	Yellowstone National Park; Rabbit Creek	Warm Creek
FECB17	5085	23	United States	Yellowstone National Park; Rabbit Creek	Warm Creek
FECB19	5091	23	United States	Yellowstone National Park; Shoshone Geyser Basin	Hot Spring
FECB21	5093-B	23	United States	Yellowstone National Park; Pott’s Geyser	Hot Spring
FECB22	5097	23	United States	Hawaii	Terrestrial (tree trunk)
FECB24	5098	23	Antarctica	McMurdo Ice Shelf; Bratina Island	Pond (freshwater)
FECB26	5099	23	Bermuda	Sommerset	Terrestrial (wooden fence)
FECB28	5102	23	Antarctica	McMurdo Ice Shelf; Bratina Island	Pond (saline)
FECB30	5108-A	23	Denmark	Limfjord Shallows, Limfjord	Marine
FECB32	6031	23	United States	Yellowstone National Park; Mammoth; Narrow Gauge	Travertine (endolithic)
FECB34	6069	23	United States	Yellowstone National Park; Lower Geyser Basin; Great Fountain	Crust
FECB36	6076	23	United States	Yellow Stone National Park; Lower Geyser Basin; Sentinel Spring	Hot Spring (silica crust)
FECB38	6083	23	United States	Yellowstone National Park; Lower Geyser Basin; Mushroom Spring	Crust
FECB52	5506	40	United States	Yellowstone National Park; Norris Geyser Basin	Terrestrial (acid crust)
FECB53	5610	40	United States	Yellowstone National Park; Sylvan Springs	Hot Spring (acid pool)
FECB58	5216	45	United States	Hunter’s Hot Spring, Oregon	Hot Spring
FECB68	5240	55	United States	Hunter’s Hot Spring, Oregon	Hot Spring

*^*a*^Co-cultures used in this work are maintained in the CCMEE and can be requested from Dr. Cady (Sherry.Cady@pnnl.gov).*

To facilitate future work, we assigned new unique FECB identifiers (FECB for Functional Encyclopedia of Cyanobacteria) to the selected co-cultures (Table 1). When requesting aliquots for future work from the CCMEE, these new FECB identifiers should be used.

FECB1 (CCMEE ID 5011) and FECB3 (CCMEE ID 5034) were collected from saline and brackish melt ponds and were dominated by phototrophic cyanobacteria previously classified as *Oscillatoria* sp. (Nadeau et al., 2001). FECB2 (CCMEE ID 5019) was collected from a freshwater pond and its composition was not investigated prior to our efforts. FECB4 (CCMEE ID 5047; AP1) and FECB5 (CCMEE ID 5049; AO21) were also isolated from freshwater and the dominant photosynthetic organisms within these samples were classified previously by 16S rRNA sequence analysis as relatives of *Pseudanabaena limnetica* and *Oscillatoria* cf. *tenuis*, respectively (Camacho et al., 1996). FECB6 (CCMEE ID 5051), FECB14 (CCMEE ID 5093; WT-97 Cal), FECB15 (CCMEE ID 5083), and FECB19 (CCMEE ID 5091; Y-97) were collected from diverse hot springs within Yellowstone National Park (YNP) (Table 1). FECB10 [CCMEE ID 5056; M88-VD (1)] was collected as epiliths (Dillon et al., 2002). FECB17 (CCMEE ID 5085; RC-97 Cal) and FECB36 (CCMEE ID 6076) were isolated from Rabbit Creek and a crust in the Sentinel Spring Meadows in YNP, respectively, and dominant phototrophs of these co-cultures were characterized previously as *Calothrix* spp. (Dillon and Castenholz, 2003). FECB22 (CCMEE ID 5097; HW-91) and FECB26 (CCMEE ID 5099; B77-scy, j) were collected from a tree trunk and a wooded fence, respectively. FECB24 (CCMEE ID 5098; AN-90) was collected from a shallow melt pond (∼10 m^2^) in the Victoria Valley, Antarctica, whereas FECB28 (CCMEE ID 5102) was collected from a saline melt pond on Bratina Island, Antarctica (Nadeau and Castenholz, 2000). FECB32 (CCMEE ID 6031), FECB34 (CCMEE ID 6069) and FECB38 (CCMEE ID 6083) were endoliths collected from subsurface (1–5 mm depths) travertine deposits in YNP (Norris and Castenholz, 2006). FECB53 (CCMEE ID 5610) was collected from Sylvan Springs in YNP. Temperature and pH at FECB53’s sampling site were determined to be 40°C and pH4, conditions which are considered to be too harsh to actively support growth of cyanobacteria, and Toplin et al. (2008) reported the thermo-acidophilic red algae *Cyanidioschyzon* as a highly abundant phototropic strain in this sample. FECB58 (CCMEE ID 5216; OH-9-45C) and FECB68 (CCMEE ID 5240; OH-2-55C) were collected from Hunter’s Hot Spring in Oregon and in 2000 Miller and Castenholz reported the isolation of several thermophilic clones belonging to the genus *Synechococcus* from these samples (Miller and Castenholz, 2000).

### Growth of Co-cultures

To obtain sufficient biomass for subsequent DNA analysis, 100 μL of each co-culture were transferred to 25 mL of sterile BG11 media (Allen and Stanier, 1968). For FECB52 and FECB53 BG11 was substituted by Cyanidium medium (Castenholz, 1981). Co-cultures were subjected to a 12 h diurnal light/dark cycle while grown over 28 days at a temperature similar to the temperature that was measured at the location where the sample was collected. Growth temperature for each sample is indicated in Table 1.

### DNA Extraction and 16S rRNA Gene Amplification

Total microbial DNA was extracted from 500 μL of each photosynthetic co-culture using the FastDNA SPIN Kit for Soil (MP Biomedical, Solon, OH, United States) according to the manufacturer’s instructions. Extracted DNA was quantified via fluorescence (Qubit; Thermo Scientific, United States) and the hypervariable V4 region of the 16S rRNA gene was amplified from extracted DNA using the primer set 515F/805R (515F: 5′-GTGCCAGCMGCCGCGGTAA-3′ and 805R: 5′-GGACTACHVGGGTWTCTAAT-3′). The forward primer included an 11 bp barcode to allow multiplexing of samples during sequencing. The barcode sequence for each sample is listed in Supplementary Table 2. Subsequent PCR reactions were performed using the 5PRIME HotMasterMix amplification mix (QIAGEN, Beverly, MA, United States) with the following PCR conditions: initial denaturation for 90 s at 94°C, followed by 30 amplification cycles (45 s at 94°C, 60 s at 60°C, and 90 s at 72°C) followed by a final extension step of 72°C for 10 min. Amplification products were cooled to 4°C. Samples were sequenced at the Department of Energy’s Joint Genome Institute (JGI; ^1^) according to JGI’s standard operating procedure using Illumina’s MiSeq platform and v3 chemistry.

### Sequence Data Analysis

Raw sequencing data were downloaded from the JGI’s Genome Portal^2^ were they are deposited and accessible under the project ID 1032475. Data were decompressed and de-interleaved using the 7-zip software^3^ and an in-house script, respectively. De-interleaved files were subsequently processed using MOTHUR version 1.38.1 (Schloss et al., 2009; Kozich et al., 2013). Paired-end reads were combined using the *make.contigs* command. Sequences with ambiguous base calls and sequences longer than 325 bp were removed using *screen.seqs*. Duplicate sequences were merged using *unique.seqs*, and the resulting unique sequences were aligned to the V4 region of the SILVA database (v123) (Quast et al., 2013). Chimeras were removed using UCHIME (Edgar et al., 2011) and quality filtered sequences were taxonomically classified at 80% confidence to the GreenGenes reference taxonomy (release gg_13_5_99) (McDonald et al., 2012). Non-prokaryotic sequences were removed and the *dist.seqs* command was used to calculate pairwise distances between the aligned sequences. The resulting pairwise distance matrix was used to cluster sequences into operational taxonomic units (OTUs) with a 97% sequence identity cut-off using UCLUST (Edgar, 2010). The most abundant sequence of each OTU was picked as the representative sequence. OTUs were taxonomically classified using the *classify.otu* command using the GreenGenes reference taxonomy (release gg_13_5_99). Shannon and Simpson estimators were calculated in MOTHUR (Schloss et al., 2009).

**FIGURE 1:**
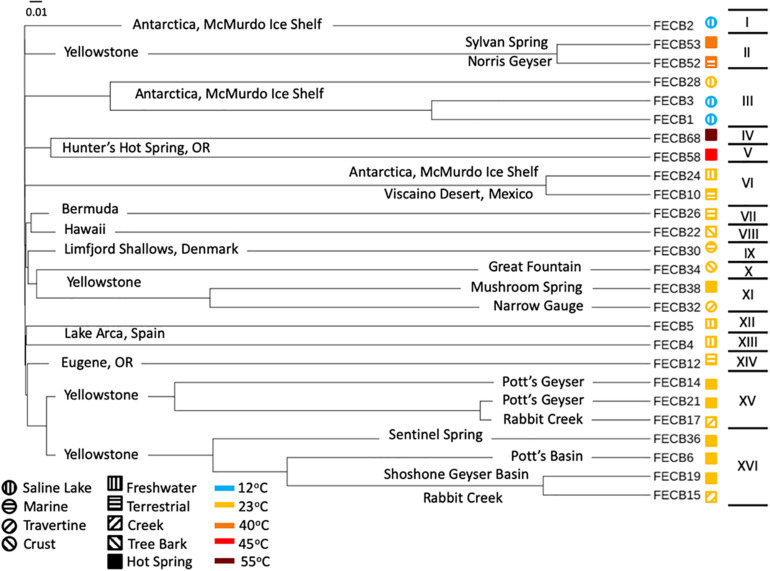
Cladogram of 16S rRNA based community composition of co-cultures under investigation. FECB identifier (sample ID) is provided for each co-culture. Sample location is indicated on the corresponding branch. Roman numerals on the right indicate the clusters identified at a branch cutoff of 0.05. Symbols (i.e., circles and squares) next to sample ID indicate habitat type and color indicates the temperatures at which sample was historically maintained in the CCMEE. Branch length indicates (number of differences/overall length of branches) between two samples.

In order to visualize the overall compositional differences between the co-cultures, an uncorrected pairwise distance matrix was generated using the *dist.seqs* command in MOTHUR and a tree was generated using *Clearcut* (version 1.0.9) (Evans et al., 2006). A cladogram from the resulting tree file was constructed and visualized using iTOL (Letunic and Bork, 2016). Cluster designations were assigned at a branch length of 0.05, with branch length indicating the (number of differences/overall length of branches) between two samples. Samples whose branches split at a distance >0.05 were considered as part of the same cluster (Figure 1).

### Availability of Data and Material

Co-cultures subject to this study are publicly available through the CCMEE upon request by contacting Sherry Cady^4^ using the corresponding FECB ID or CCMEE ID (Table 1). Co-cultures can also be obtained from the Hess Lab at UC Davis. Sequences generated during this project have been deposited and are publicly available at NCBI’s SRA under the BioProject ID PRJNA401502. All other data is included in this published article and its Supplementary Information files.

The CCMEE is now maintained at the Northwest National Laboratory by Dr. Cady. The CCMEE is comprised of >1,200 co-cultures, including the co-cultures that were studied in the work presented here, and has been established to provide a valuable resource to the scientific community. Cultures that are part of the CCMEE can be requested from Sherry Cady^1^.

## Results and Discussion

### Prokaryotic Diversity and Eukaryotic Population of Co-cultures

A total of 3,357,905 raw reads [mean (SD) = 129,150 (±15,845) reads per sample] were generated from the V4 region of the 16S rRNA gene (Table 2). Quality filtering removed ∼3.8% (±0.57%) of the raw reads from each sample due to insufficient quality. The remaining reads were assigned to a total of 5,785 distinct Operational Taxonomic Units (OTUs) based on 97% sequence identity (Supplementary Table 3).

**TABLE 2:** Read statistics and diversity Index for co-cultures investigated in this study.

**Sample ID**	**Total raw reads**	**Total quality filtered reads**	**Total OTUs observed**	**OTUs recruiting >0.1% of reads**	**% Reads represented by OTUs recruiting >0.1% of reads**	**% reads recruited by dominant OTU**	**Inverse Simpson index**	**Shannon index**
FECB1	154,251	139,666	349	13	99.6%	47%	3.23	1.49
FECB2	128,172	116,166	197	3	99.8%	51%	2.04	0.77
FECB3	139,819	128,879	274	8	99.6%	80%	1.52	0.76
FECB4	94,960	84,983	288	17	99.4%	44%	3.92	1.77
FECB5	122,414	108,882	401	18	99.4%	40%	4.63	2.00
FECB6	153,355	136,199	549	28	99.4%	29%	6.5	2.39
FECB10	114,324	102,534	299	10	99.7%	72%	1.78	0.96
FECB12	151,629	141,953	335	19	99.5%	72%	1.87	1.23
FECB14	145,839	98,588	206	4	99.6%	67%	1.83	0.75
FECB15	105,256	96,030	278	9	99.6%	63%	2.18	1.14
FECB17	109,474	97,391	317	7	99.5%	59%	2.47	1.33
FECB19	118,003	103,477	300	10	99.5%	47%	3.27	1.50
FECB21	127,371	113,281	401	15	99.2%	58%	2.38	1.27
FECB22	129,934	117,651	399	23	99.4%	40%	4.46	2.08
FECB24	115,275	93,362	265	12	99.9%	65%	2.12	1.17
FECB26	121,303	103,597	265	16	99.5%	63%	2.29	1.32
FECB28	152,974	134,318	332	10	99.4%	37%	3.22	1.36
FECB30	140,681	118,256	546	22	98.7%	33%	4.61	2.00
FECB32	116,889	72,130	336	29	98.3%	19%	9.24	2.68
FECB34	138,723	125,710	288	14	99.9%	76%	1.69	1.11
FECB36	137,395	124,885	360	12	99.6%	52%	2.38	1.16
FECB38	139,001	124,379	479	17	99.1%	32%	5.22	2.01
FECB52	117,261	75,433	367	7	99.9%	71%	1.85	1.02
FECB53	119,380	86,565	303	5	99.1%	91%	1.21	0.44
FECB58	135,683	124,119	351	13	99.3%	38%	3.65	1.61
FECB68	128,539	114,032	402	11	99.7%	26%	5.27	1.90
Total	3,357,905	2,882,466	N/A	N/A	N/A	N/A	N/A	N/A
Average	129,150	110,864	342	14	99%	51%	N/A	N/A
Stdev	15,845	19,275	87.2	6	0%	17%	N/A	N/A

To estimate the microbial diversity within each sample, rarefaction analyses were performed (Supplementary Figure 2) and diversity indices were calculated (Table 2). The Inverse Simpson index (Simpson, 1949; Morris et al., 2014) of the samples ranged between 1.21 and 9.24 with the lowest and highest indices calculated for FECB53 and FECB32, respectively (Table 2), illustrating that co-cultures investigated during this project represented co-cultures from a wide range of diversity. Not surprisingly, the diversity in the co-cultures under investigation appeared to be negatively correlated with the proportion of reads recruited by the dominant OTU of each sample (Pearson *r* = −0.8806; *p* < 0.01). Although samples ranked slightly differently based on their diversity, when diversity was calculated using the Shannon index (Kim et al., 2017), the overall trend remained the same (Table 2).

The presence of eukaryotic microorganisms in each co-culture was estimated using mitochondrial reads resulting from the 16S rRNA sequencing (Supplementary Table 4). FECB52 had the greatest percentage of mitochondrial DNA at 0.29% of total reads. FECB2, FECB4, FECB6, FECB14, FECB17, FECB26, FECB30, and FECB68 all contained no mitochondrial reads. Average mitochondrial reads as a percentage of all reads averaged 0.02% across all 26 samples.

### Ubiquity of Cyanobacteria and Proteobacteria Within Photosynthetic Co-cultures

**FIGURE 2:**
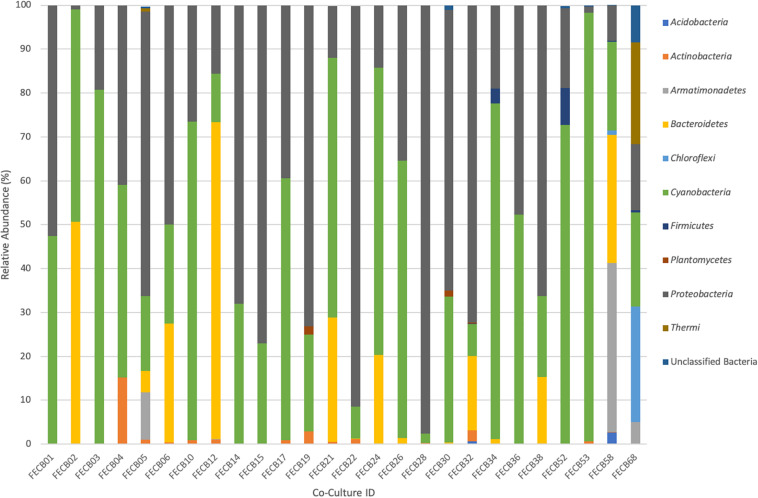
Relative abundance of phyla associated with phototrophic co-cultures. 16S rRNA based community profile. Only phyla recruiting >0.1% of the reads in at least one of the co-cultures are shown.

While the microbial communities of the co-cultures under investigation varied greatly, cyanobacteria and proteobacteria co-occurred in all 26 of the community assemblages. Community composition analysis revealed that each of the co-cultures contained at least one OTU [mean (SD) = 2 (±1.23)] that recruited (>0.1% of the co-culture specific reads and that was classified as Cyanobacteria (Table 3). The only other phylum present in each of the individual 26 co-cultures and represented by at least one OTU recruiting (>0.1% of the reads was the *Proteobacteria* phylum (Table 3). In contrast, only three samples, namely FECB5, FECB30, and FECB68, contained OTUs that recruited >0.1% of the sample specific reads and that could not be classified at the phylum level or at a higher taxonomic resolution (Table 3). It is possible that the relatively high abundance of non-classified phyla might contribute to the separation of these samples into distinct clusters (i.e., cluster XII, IX, and IV; Figure 1). In addition to their ubiquity, *Cyanobacteria* and *Proteobacteria* also recruited the majority of the reads in all but four (i.e., FECB2, FECB12, FECB58, and FECB68) of the samples under investigation (Figure 2 and Supplementary Table 5). In FECB2 and FECB12 the majority of the reads were recruited by OTUs classified as members of the phylum *Bacteroidetes* (recruiting 50.6 and 72% of the reads, respectively), whereas within FECB58 and FECB68, *Armatimonadetes* (38.3%) and *Chloroflexi* (25.9%) were identified as the most abundant phyla (Figure 2 and Supplementary Table 5). The fact that these samples were dominated by phyla other than the *Cyanobacteria* or *Proteobacteria* may also help to explain why these samples form distinct clusters (cluster I, XIV and V, IV, respectively; Figure 1).

**TABLE 3:** Count and phylogenetic classification of identified OTUs at the phylum level.

**Phylum**	**FECB 1**	**FECB 2**	**FECB 3**	**FECB 4**	**FECB 5**	**FECB 6**	**FECB 10**	**FECB 12**	**FECB 14**	**FECB 15**	**FECB 17**	**FECB 19**	**FECB 21**	**FECB 22**	**FECB 24**	**FECB 26**	**FECB 28**	**FECB 30**	**FECB 32**	**FECB 34**	**FECB 36**	**FECB 38**	**FECB 52**	**FECB 53**	**FECB 58**	**FECB 68**
Cyanobacteria	2	1	2	1	1	3	1	2	2	2	1	2	6	5	1	1	1	2	3	1	2	2	3	1	1	2
Proteobacteria	10	1	5	13	11	18	7	12	2	7	5	5	6	16	10	14	8	16	20	11	9	14	3	2	6	2
Actinobacteria	1	0	1	3	1	1	2	3	0	0	1	2	1	2	0	0	1	0	3	0	1	0	0	2	0	0
Bacteroidetes	0	1	0	0	1	6	0	1	0	0	0	0	2	0	1	1	0	1	1	1	0	1	0	0	1	0
Armatimonadetes	0	0	0	0	1	0	0	1	0	0	0	0	0	0	0	0	0	0	0	0	0	0	0	0	1	1
Unclassified Bacteria	0	0	0	0	1	0	0	0	0	0	0	0	0	0	0	0	0	1	0	0	0	0	0	0	0	2
Firmicutes	0	0	0	0	0	0	0	0	0	0	0	0	0	0	0	0	0	0	0	1	0	0	1	0	1	1
Thermi	0	0	0	0	1	0	0	0	0	0	0	0	0	0	0	0	0	0	0	0	0	0	0	0	0	1
Chloroflexi	0	0	0	0	1	0	0	0	0	0	0	0	0	0	0	0	0	0	0	0	0	0	0	0	2	2
Acidobacteria	0	0	0	0	0	0	0	0	0	0	0	0	0	0	0	0	0	0	1	0	0	0	0	0	1	0
Planctomycetes	0	0	0	0	0	0	0	0	0	0	0	1	0	0	0	0	0	2	1	0	0	0	0	0	0	0
Total OTU count	13	3	8	17	18	28	10	19	4	9	7	10	15	23	12	16	10	22	29	14	12	17	7	5	13	11

*Only OTUs recruiting >0.1% of the co-culture specific reads are shown.*

### Firmicutes Dominate Photosynthetic Co-cultures From Hot Springs

*Firmicutes* abundances calculated for co-cultures from hot spring samples were higher compared to those calculated for co-cultures from other environments. OTUs assigned to the *Firmicutes* phylum were detected above the applied cut-off level of 0.1% in only five of the twenty-six co-cultures under investigation (Table 3). Interestingly, these samples (i.e., FECB32, FECB34, FECB52, FECB58, and FECB68) are co-cultures collected from hot springs or from deposits within hot springs, with FECB52, FECB58, and FECB68 being maintained in culture at temperatures >40°C. OTU000073 (classified as *Alicyclobacillus tolerans*), OTU00082 (classified as members of the genus *Paenibacillus*), OTU000154 (classified as *Geobacillus vulcani*), and OTU000158 (classified as a member of the *Bacillaceae* family) recruited 5.9, 3.4, 0.5, and 0.4% of the reads generated from FECB52, FECB34, FECB68, and FECB58, respectively (Supplementary Table 3). *Alicyclobacillus tolerans* and *Geobacillus vulcani* have been described previously as aerobic spore-forming thermophiles and have been isolated from lead–zinc ores (Karavaiko et al., 2005) and hot springs (Nazina et al., 2004) located in Russia, respectively. Members of the genus *Paenibacillus* have been isolated from a wide variety of environments and some *Paenibacillus* species have been found to promote crop growth directly via biological nitrogen fixation, phosphate solubilization, production of the phytohormone indole-3-acetic acid; and they have been identified as a potential source of novel antimicrobial agents (Grady et al., 2016).

### Photosynthetic Co-cultures From Antarctica and YNP to Study Adaptation to Increased Radiation, Low Temperatures and Oligotrophic Growth Conditions

Microbial adaptation to extreme environments and the molecular framework that enable microorganisms to survive and thrive in the presence of increased rates of radiation, low temperatures and in the absence of nutrients has fascinated the scientific community for decades and remains poorly understood. In an attempt to provide a better basis of the taxonomic make-up of co-cultures that were collected from ecosystems that are characterized by these extremes, we included co-cultures from Antarctica and YNP in this study (Table 1). OTU-based comparison of Antarctica and YNP co-cultures revealed between 197 (FECB2) and 549 (FECB6) distinct OTUs [mean (SD) 342 (87.2) OTUs] based on 97% sequence similarity (Table 2). The number of OTUs that recruited >0.1% of all reads ranged from 3 to 29 OTUs, with FECB2 and FECB32 having the lowest and highest OTU count, respectively (Table 2). FECB2 was dominated by an OTU classified as *Hymenobacter*, which recruited all *Bacteroidetes*-specific reads generated from this sample (Tables 3, 4). The genus *Hymenobacter* contains several pigmented bacteria that have been isolated from Antarctica and have been reported to possess increased resistance to radiation (Oh et al., 2016; Marizcurrena et al., 2017), which might explain their increased abundance in FECB2, a co-culture isolated from an environment known to possess increased levels of UV radiation. Taking this into consideration, FECB2 and its individual community members could be a potential target for future studies to enhance our understanding of processes that infer resistance to radiation and DNA damage. The second most abundant OTU in FECB2, recruiting 48% of the generated samples, was classified as *Phormidium* sp. (Supplementary Table 3), a cyanobacterial genus that has been reported to dominate aquatic microbial mats from Antarctica (Jungblut et al., 2005; Strunecky et al., 2012). Representative isolates from this genus have been proposed previously as cost-effective options for industrial carotenoid production (Shukla and Kashyap, 2003), suggesting that FECB2 may hold the potential for this process.

**TABLE 4:** Taxonomy relative abundance of dominant OTU identified in each co-culture.

**Sample**	**Taxonomic assignment**	**% Reads recruited**
FECB1	*Cyanobacteria; Oscillatoriophycideae; Oscillatoriales; Phormidiaceae; Phormidium; pseudopriestleyi*	47
FECB2	*Bacteroidetes; Cytophagia; Cytophagales; Cytophagaceae; Hymenobacter*	51
FECB3	*Cyanobacteria; Oscillatoriophycideae; Oscillatoriales; Phormidiaceae; Phormidium; pseudopriestleyi*	80
FECB4	*Cyanobacteria; Oscillatoriophycideae; Oscillatoriales; Phormidiaceae; Planktothrix*	44
FECB5	*Proteobacteria; Alphaproteobacteria; Sphingomonadales; unclassified*	40
FECB6	*Proteobacteria; Gammaproteobacteria; Pseudomonadales; Pseudomonadaceae; Pseudomonas*	29
FECB10	*Cyanobacteria; Nostocophycideae; Nostocales; Nostocaceae; unclassified*	70
FECB12	*Bacteroidetes; Saprospirae; Saprospirales; Chitinophagaceae; Sediminibacterium*	72
FECB14	*Proteobacteria; Alphaproteobacteria; Rhodospirillales; Rhodospirillacaea; Phaeospirillum; fulvum*	67
FECB15	*Proteobacteria; Gammaproteobacteria; Pseudomonadales; Pseudomonadaceae; Pseudomonas*	63
FECB17	*Cyanobacteria; Nostocphycideae; Stigonematales; Rivulariaceae; Rivularia*	59
FECB19	*Proteobacteria; Gammaproteobacteria; Pseudomonadales; Pseudomonadaceae; Pseudomonas*	47
FECB21	*Cyanobacteria; Nostocophycideae; Stigonematales; Rivulariaceae; Rivularia*	58
FECB22	*Proteobacteria; Alphabacteria; Caulobacterales; Caulobacteraceae; Mycoplana*	40
FECB24	*Cyanobacteria; Nostocphycideae; Nostocales; Nostocaceae; unclassified*	65
FECB26	*Cyanobacteria; Nostocophycideae; Nostocales; unclassified*	63
FECB28	*Proteobacteria; Gammaproteobacteria; Altermonadales; Alteromonadaceae; Marinobacter; hydrocarbonoclasticus*	37
FECB30	*Cyanobacteria; Oscillatoriophycideae; Oscillatoriales; Phormidiaceae; Geitlerinema*	33
FECB32	*Proteobacteria; Alphaproteobacteria; Sphingomonadales; Sphingomonadaceae; Sphingopyxis;alaskensis*	19
FECB34	*Cyanobacteria; Nostocophycideae; Nostocales; Nostocaceae; unclassified*	76
FECB36	*Cyanobacteria; Oscillatoriophycideae; unclassified*	52
FECB38	*Proteobacteria; Gammaproteobacteria; Xanthomonadales; Xanthomonadaceae; Stenotrophomonas; geniculata*	32
FECB52	*Cyanobacteria; unclassified*	71
FECB53	*Cyanobacteria; unclassified*	91
FECB58	*Armatimonadetes; OS-L; unclassified*	38
FECB68	*Chloroflexi; Chloroflexi; Chloroflexales; Chloroflexaceae; Chloroflexus*	26

FECB32 is a mixed culture isolated from an ancient travertine at Mammoth in YNP. Our analysis indicated that FECB32 contained 29 OTUs that each accounted for >0.1% of the reads generated (Table 2). Fifteen of these OTUs recruited >1% of all reads and four OTUs collectively accounted for ∼60% of the reads generated from this co-culture (Supplementary Table 5). These four OTUs were classified as *Sphingopyxis alaskensis, Chelativorans* sp., and as members of the *Chitinophagaceae* and *Comamonadaceae families*, recruiting ∼19, 13, 17, and 11% of the reads, respectively (Supplementary Tables 3,4). *S. alaskensis* is a Gram-negative bacterium found in relatively high abundance in oligotrophic regions of the ocean (Vancanneyt et al., 2001; Cavicchioli et al., 2003) and it has been studied in great detail as a model system for marine bacteria, specifically to understand microbial adaptation to cold or oligotrophic environments (Lauro et al., 2009; Ting et al., 2010). The *Chitinophagaceae* family contains a wide phylogenetic diversity with many of its members being mesophilic. However, *Chitinophagaceae* have been reported to grow optimally at temperatures of 55°C and higher (Anders et al., 2014; Hanada et al., 2014).

### Photosynthetic Co-cultures Containing the Deep-Branching Candidate Phylum Melainabacteria

Extreme environments similar to those on early Earth are often proposed to hold critical information about the historical progression of life on our planet and a niche that encompasses those physical stresses is the endolithic environment of rocks (Norris and Castenholz, 2006). Phylogenetic analysis of the heterotrophic population associated with FECB32, which was isolated from travertine deposited by hot springs in YNP, found that sequences from MLE-12 (OTU000109) recruited ∼2% of the sample specific sequences (Supplementary Table 3). This rendered MLE-12, previously assigned to the deep-branching candidate phylum *Melainabacteria* (Di Rienzi et al., 2013), as the eleventh most abundant organism in this photosynthetic co-culture. It has been proposed previously that *Melainabacteria*, which is commonly found in aquatic habitats, separated from the cyanobacteria before the latter acquired photosynthetic capabilities (Di Rienzi et al., 2013). Hence FECB32 might be a particularly valuable co-culture to generate new insights into the evolution of and relationship between the phylogenetically closely related *Cyanobacteria* and *Melainabacteria*.

Interestingly, OTU000109 was also detected in FECB36 and FECB38 (Supplementary Table 3), although at significantly lower abundance (<0.001%). FECB36 and FECB38 were similar to FECB32 in that they were isolated from sites in YNP. Interestingly, FECB32 and FECB38 cluster together (cluster IX) suggesting similar overall microbial community profiles, but separately from FECB36 (Figure 1). The only additional samples that contained OTUs classified as *Melainabacteria*, recruiting >0.1% of the generated reads, were FECB58 and FECB68 with ∼0.9 and ∼0.2% of their reads to this deeply branched phylum, respectively (Supplementary Table 5). It seems noteworthy that FECB58 and FECB68 were also isolated from hot springs and clustered closely together based on their overall microbiome composition (Clusters V and IV, respectively; Figure 1).

### The McMurdo Dry Valley Lake System, a Physically Highly Stable Lacustrine System

The McMurdo Dry Valley (MDV) is one of the most extreme deserts on Earth, and although the importance of microbial communities for biogeochemical cycles of this region is widely accepted, the microbial ecology of the MDV remains poorly understood (Chan et al., 2013). FECB3, originating from a brackish pond on Bratina Island, was dominated by OTU000003, which recruited 80.3% of all reads (Supplementary Table 5). OTU000003 was classified as the cyanobacterium *Phormidium pseudopriestleyi*, previously reported to dominate microbial mats of the anoxic zone of Lake Fryxell, Antarctica (Jungblut et al., 2015). The second and third most abundant OTUs in FECB3 were OTU000015 and OTU000061, respectively (Supplementary Table 5). Both OTU000015 and OTU000061 were classified as Rhodobacteriaceae and recruited 9.2 and 8.2% of the reads generated for FECB3. Whereas a taxonomic classification of OTU000015 was not possible beyond the family level, OTU000061 was classified as *Paracoccus marcusii*, a Gram-negative organism that displays a bright orange color due to the synthesis of carotenoids such as astaxanthin (Harker et al., 1998).

### Photosynthetic Co-cultures From Hunter’s Hot Spring, Oregon

FECB58 and FECB68 were both isolated from Hunters Hot Spring in Oregon, United States and they shared similar microbial community members. Despite their similar community profile, abundances of the dominant OTUs associated with these two hot spring co-cultures were remarkably different. FECB58 was dominated by three OTUs (OTU000014, OTU000024, and OTU000033). OTU000014 was classified as OS-L, an uncultured representative of the phylum *Armatimonadetes*, OTU000024 which was classified as belonging to the *Bacteroidetes* phylum, and OTU000033 which was classified as *Thermosynechococcus*. These OTUs contributed 38, 29, and 20% of the reads generated from FECB58, respectively. Whereas OTU000014 recruited ∼4.9% of all reads generated from FECB68, representing the sixth most abundant OTU in the FECB68 community, OTU000024 and OTU000033 were only present at an abundance <0.0001% in FECB68 (Supplementary Table 3).

FECB68 was dominated by 6 OTUs (i.e., OTU000028, OTU000030, OTU000036, OTU000049, OTU000065, and OTU000014) recruiting ∼25.7, 23.1, 20.4, 14.3, 7.6, and 4.9% of the reads, respectively. OTU000028 was classified as belonging to the genus *Chloroflexus*, whereas OTU000030 and OTU000036 were classified as representative of the genus *Meiothermus* and *Gloeobacter*, respectively. *Chloroflexus* is an anoxygenic phototrophic bacterium that grows at temperatures up to 70°C (Castenholz, 2015) and forms yellow-orange-greenish mats in association with cyanobacteria (Hanada, 2014). Members of the cyanobacterial genus *Gloeobacter* lack thylakoids, and have been proposed to host the earliest ancestors, or a missing link, in the cyanobacteria lineage (Saw et al., 2013). Thus, FECB68 offers a unique opportunity to investigate interspecies interaction between a member of these basal cyanobacteria and the thermophilic phototroph *Chloroflexus*, represented by OTU000028 in this co-culture. As outlined in a recent review by Castenholz (2015), Hunter’s Hot Spring located in Oregon is one of the most studied hot springs in the world and a large repertoire of work has been conducted on this habitat over the last 40 years. However, most of this work was performed prior to the advent of recent molecular and -omics techniques.

### Photosynthetic Co-cultures From Lignocellulosic Surfaces With Potential to Fix Nitrogen and Degrade Aromatic Compounds

FECB22 and FECB26 are mesophilic co-cultures collected from similar habitats (i.e., from tree bark and a wooden fence) from two locations (i.e., Hawaii and Bermuda) approximately 9,000 kilometers apart from each other (Supplementary Figure 1 and Table 1). Diversity index calculation placed these two samples in the mid-range of the diversity spectrum of the 26 co-cultures analyzed for this study. The inverse Simpson and Shannon index was calculated at 4.46 and 2.08 for FECB22 and 2.29 and 1.32 for FECB26, respectively (Table 2). Within FECB22, 23 OTUs were identified as individually recruiting more than 0.1% of the generated reads. In contrast, FECB26 contained only 16 OTUs that recruited more than 0.1% of the reads each (Supplementary Table 5). FECB22, scraped from tree bark in Hawaii, was dominated by 11 OTUs, each recruiting (>1% of the reads. The most abundant OTU (OTU000017) was classified as a member of the Mycoplana, a genus that contains bacteria capable of aromatic compound degradation (Urakami et al., 1990), and it recruited 40.2% of the reads. OTU000042 (classified as *Rhizobium leguminosarum*), OTU000045 (classified as *Acetobacteraceae*), and OTU000072 (classified as *Cyanobacteria*), were the next most abundant OTUs, recruiting 17.1, 16.3, and 5.5% of the reads generated from FECB22, respectively. *Rhizobium leguminosarum* is a well-studied α-proteobacterium capable of N_2_-fixation and “rhizobia” have been suggested repeatedly to facilitate more sustainable agricultural practices through their symbiosis with legumes, reducing the need for nitrogen fertilizer (Marek-Kozaczuk et al., 2017). It remains to be seen if OTU000042 provides *N*_2_ to the other organisms in this co-culture or if it consumes all of the fixed *N*_2_ itself. *Acetobacteraceae* are α-proteobacteria often associated with low pH environments and are known for their ability to efficiently synthesize biological cellulose (Rozenberga et al., 2016; Semjonovs et al., 2017). Furthermore, *Acetobacteraceae* have been reported before as some of the dominant players in photosynthetic consortia during soil formation (Mapelli et al., 2011). It would be interesting to explore the agricultural and chemical potential of a minimalistic co-culture composed of the four OTUs (i.e., OTU000017, OTU000042, OTU000045, and OTU000072) that dominated FECB22, as they may combine the ability to degrade aromatic compounds and synthesize cellulose while removing nitrogen from the atmosphere. FECB26, on the other hand, was dominated by OTU000010, which recruited 63.2% of the reads generated and it was identified as an unclassified member of the *Nostocales*; a phylogenetic group known for their functional and morphological diversity. Members of the *Sphingomonadaceae* (i.e., OTU000041 and OTU000062), phototropic α-proteobacteria often found in high abundance in environments previously thought to support mostly the growth of cyanobacteria (Tahon and Willems, 2017), contributed to a total of 25.6% of the generated reads. Most interestingly, OTU000017 was also detected within FECB26 recruiting ∼1.6% of the reads. It is possible that OTU000017 facilitates a metabolic reaction in which aromatic compounds typically associated with the decomposition of woody material under aerobic conditions are utilized.

## Conclusion

Culture collections can provide easy access to biological samples without the need for extensive resources by the requesting individual, subsequently facilitating new studies and ultimately advancing our understanding of phylogenetic and functional biodiversity. While these collections present increased access to typically hard to acquire samples, there is lost diversity due to cultivation bias, but it remains to be understood exactly how prevalent and consistent the loss of diversity is sample to sample. Although care is taken to mimic the native environmental conditions of each sample in the cultivation process, there are real world factors that either cannot be mimicked in a lab setting or are unknown to researchers. More work is needed to assess this cultivation bias and to develop techniques to minimize the effects. Although some of the diversity of the original microbial community might have been lost due to a cultivation bias, the 16S rRNA based community fingerprints of the 26 photosynthetic co-cultures described here provide a first in-depth glimpse into the taxonomic and functional diversity of communities from extreme environments that were considered for a long time as too harsh to support the growth of complex microbial communities. The extreme conditions that are associated with the habitats from which these co-cultures were collected offer the unique opportunity to study the molecular mechanisms that support the growth of these extremophilic co-cultures and their role in global carbon and nitrogen cycling. Co-cultures from the CCMEE, and data presented here, also provide a first opening to enhance our understanding of the origin of oxygenic photosynthesis and aerobic respiration in *Cyanobacteria*, an area that is currently still poorly understood (Soo et al., 2017). Furthermore, an in-depth understanding of these co-cultures holds the potential to discover novel microbial proteins that might render current agricultural, industrial and medical processes more economical and sustainable, for example by promoting or inhibiting plant and microbial growth.

The heterogeneity of the physical parameters reported for the sites where the samples presented in this work were collected, highlights a major challenge (i.e., standardization of protocols) associated with environmental samples and their corresponding metadata (i.e., data describing conditions at each sampling site), specifically when collected during independent sampling efforts. Fortunately, with recent advances in data technologies, the task of data acquisition and dissemination has become less of a challenge. In order to make the best use of these technologies defining a set of minimal information parameters to be recorded during the collection of an environmental sample is of great importance. Similar efforts have been successfully implemented by the Genomic Standards Consortium (GSC) for microbial genomes and metagenomes in the form of the “minimum information about a genome sequence” (MIGS) (Field et al., 2008) and are enforced when describing a novel microbial species (Kampfer et al., 2003).

The identification of Minimum Information about a Co-Culture Sample (MICCS) would be a significant step in standardizing sample acquisition and maintenance, increasing the value of current and future microbial samples collected from the environment. Developing MICCS and applying them to co-cultures currently available from existing culture depositories is beyond the scope of the work presented here, but we hope that the results presented here will contribute to the initiation of this process and stimulate broad involvement and support from the scientific community and various funding agencies.

In summary, we encourage the scientific community to take advantage of the CCMEE and the data we generated during this pilot study. Both data and samples from which these data were generated are publicly available from the CCMEE for further in-depth analyzes and investigations. Future work which might provide a more detailed picture of the microbe-microbe interactions in these co-cultures and their role in the global carbon and nitrogen cycle.

## Data Availability Statement

The datasets presented in this study can be found in online repositories. The names of the repository/repositories and accession number(s) can be found below: https://www.ncbi.nlm.nih.gov/genbank/, PRJNA401502.

## Author Contributions

CS, CB, RC, DC, MH, and ST wrote the manuscript. RC and MH designed the experiment. EH and MH performed the experiment. MB, TG, MH-S, EH, MH, NS, and ST generated the data. MB, CS, CB, MC, DC, JG, TG, MH-S, EH, MH, and NS analyzed the data. All authors contributed to the article and approved the submitted version.

## Conflict of Interest

EH was employed by the company Bayer. DC was employed by the company Greenlight Biosciences. The remaining authors declare that the research was conducted in the absence of any commercial or financial relationships that could be construed as a potential conflict of interest.

## Footnotes

1 http://www.jgi.doe.gov

2 http://genome.jgi.doe.gov/

3 http://www.7-zip.org

4 http://sherry.cady@pnnl.gov
