# Supplementary material for: Phototrophic Co-cultures From Extreme Environments: Community Structure and Potential Value for Fundamental and Applied Research
Source: Front Microbiol. 2020 Nov 6;11:572131. doi: 10.3389/fmicb.2020.572131 (PMC7677454; doi:10.3389/fmicb.2020.572131)
Supplement: Supplementary file 1 [file Data_Sheet_1.PDF]

SUPPLEMENTARY INFORMATION

Title: Phototrophic co-cultures from extreme environments: community structure and potential value for fundamental and applied research

Claire Shaw[1], Charles Brooke[1], Erik Hawley[2], Morgan P. Connolly[3], Javier A. Garcia[4], Miranda Harmon-Smith[5], Nicole Shapiro[5], Michael Barton[5], Susannah G. Tringe[5], Tijana Glavina del Rio[5], David E. Culley[6], Richard Castenholz[7] and *Matthias Hess[1]

[1] Systems Microbiology & Natural Products Laboratory, University of California, Davis, CA
[2] Bayer, Pittsburg, PA
[3] Microbiology Graduate Group, University of California, Davis, CA
[4] Biochemistry, Molecular, Cellular, and Developmental Biology Graduate Group, University of California, Davis, CA
[5] Department of Energy, Joint Genome Institute, Berkeley, CA
[6]Greenlight Biosciences, Medford, MA
[7] University of Oregon, Eugene, OR

[1] Systems Microbiology & Natural Products Laboratory, University of California, Davis, CA
[2] Bayer, Pittsburg, PA
[3] Microbiology Graduate Group, University of California, Davis, CA
[4] Biochemistry, Molecular, Cellular, and Developmental Biology Graduate Group, University of California, Davis, CA
[5] Department of Energy, Joint Genome Institute, Berkeley, CA
[6]Greenlight Biosciences, Medford, MA
[7] University of Oregon, Eugene, OR

***Corresponding author:**

Matthias Hess
University of California, Davis
2251 Meyer Hall
Davis, CA 95616, USA
P (530) 530-752-8809
F (530) 752-0175
mhess@ucdavis.edu

SUPPLEMENTAL FIGURES

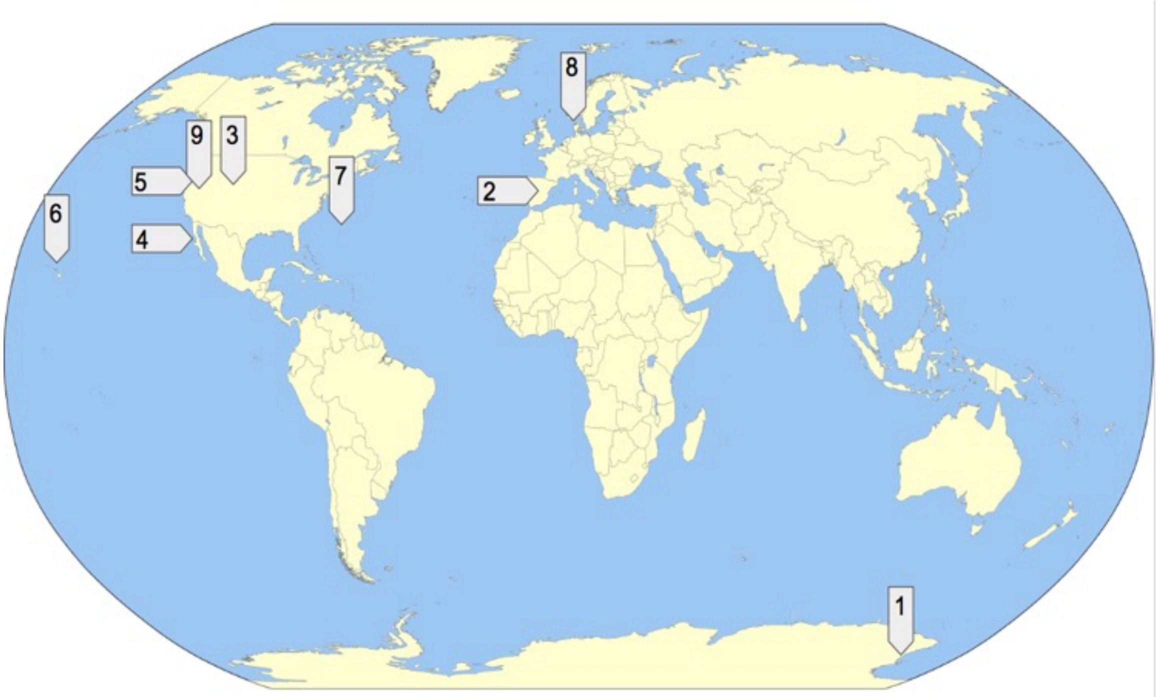

Figure S1: Geographical locations of co-cultures analyzed. 1.) Antarctica. McMurdo Ice Shelf; Bratina Island; 2.) Spain. Lake Arcas; 3.) USA. Yellowstone National Park; 4.) Mexico. Vizcaino Desert; 5.) USA. Eugene, Oregon. 6.) USA. Hawaii; 7.) Bermuda, Somerset; 8.) Denmark. Limfjord Shallows; 9.) USA. Hunter's Hot Spring, Oregon. (Map downloaded and adapted from https://commons.wikimedia.org/wiki/File:ColoredBlankMap-World-10E.svg#file)

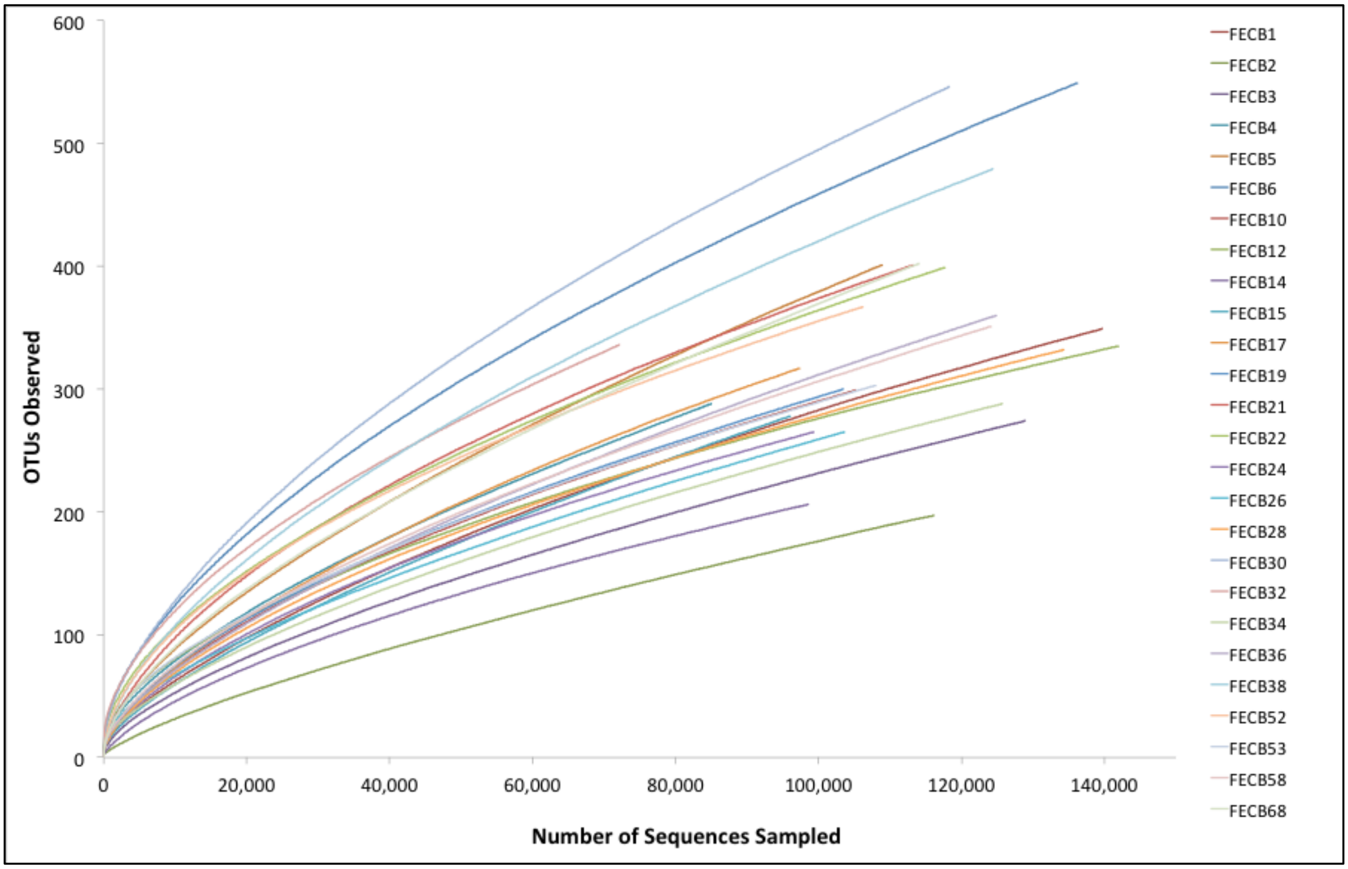

Figure S2: Rarefaction curves calculated from 16S iTag data of photosynthetic co-cultures from extreme environments.

TABLE LEGENDS

Table S1: DNA Barcodes used in this study.

Table S2: OTU classification and abundance.

Table S3: Relative abundance of phyla within the co-cultures of this study. Only phyla recruiting >1% of the reads in at least one of the co-cultures are shown.

Table S4: Relative abundance of OTUs within the co-cultures of this study. Only OTUs recruiting >0.1% of the co-culture specific reads are shown.
