# Supplementary figures and images for: Phototrophic Co-cultures From Extreme Environments: Community Structure and Potential Value for Fundamental and Applied Research
Source: Front Microbiol. 2020 Nov 6;11:572131. doi: 10.3389/fmicb.2020.572131 (PMC7677454; doi:10.3389/fmicb.2020.572131)

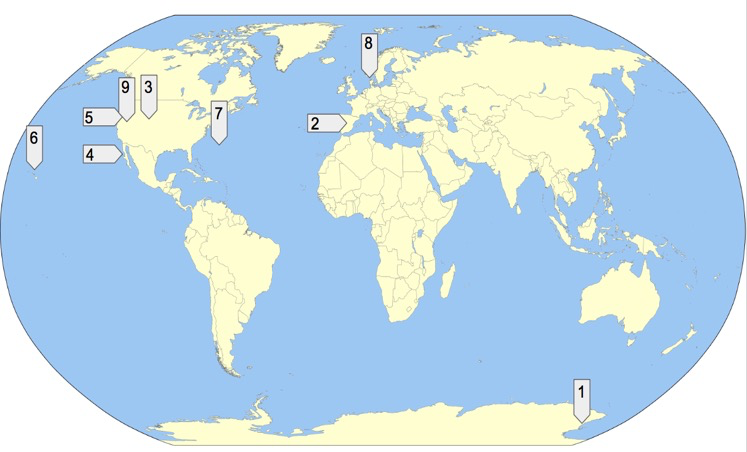

Supplement: Supplementary file 2 [file Image_1.TIFF]

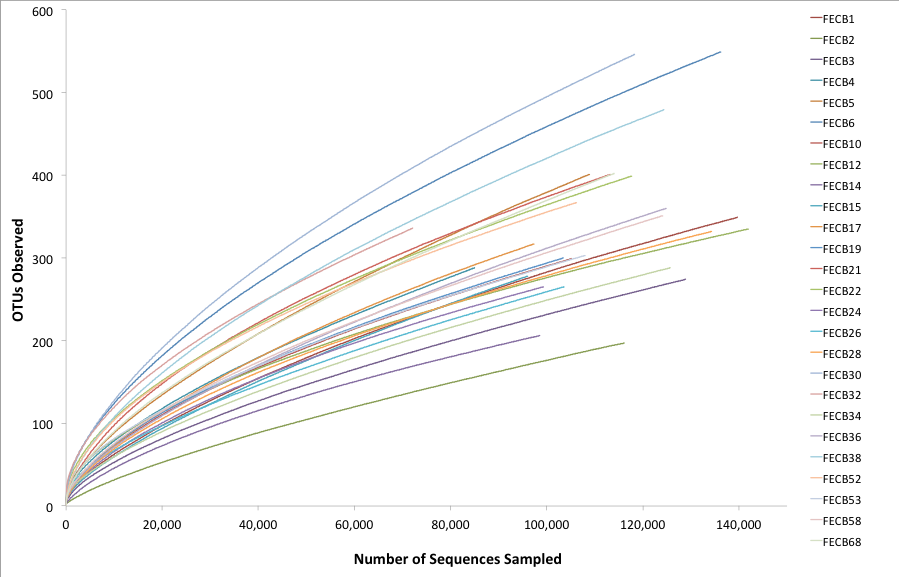

Supplement: Supplementary file 3 [file Image_2.TIFF]
